# Cerebral ischemia after treatment of posterior communicating artery aneurysms: clipping versus coiling

**DOI:** 10.1186/s12883-022-02962-1

**Published:** 2022-11-17

**Authors:** Yuankun Cai, Tingbao Zhang, Jingwei Zhao, Guo Li, Jincao Chen, Wenyuan Zhao, Nanxiang Xiong

**Affiliations:** Department of Neurosurgery, Zhongnan Hospital of Wuhan University, Wuhan, P.R. China

**Keywords:** Aneurysm, Clipping, Cerebral ischemia, Coiling, Posterior communicating artery

## Abstract

**Objection:**

This study aimed to compare the incidence of cerebral ischemia and outcomes between surgical clipping and endovascular coiling in patients with posterior communicating artery (PCoA) aneurysms.

**Methods:**

Clinical and imaging data of patients with at least one PCoA aneurysm who underwent surgical clipping or endovascular coiling in our institution from January 2017 to December 2019 were analyzed.

**Results:**

Three hundred sixty-three aneurysms in 353 patients were included for analysis, 257 in the clipping group, and 106 in the coiling group. The groups did not differ in terms of baseline characteristics. The incidence of postoperative cerebral ischemia (23.35% vs. 11.32%, *p* = 0.029) was higher in the clipping group. The proportion of patients with a modified Rankin Scale score ≥ 2 was significantly higher in the clipping group at discharge (35.80% vs. 15.09%; *p* < 0.05) but not six months after discharge (15.56% vs. 8.49%; *p* > 0.05). In the clipping group, the mean age was significantly higher in patients who developed cerebral ischemia than in those who did not. In the coiling group, modified Fisher grade and incidence of fetal PCoA were significantly higher in patients who developed ischemia.

**Conclusion:**

The incidence of postoperative cerebral ischemia was higher after PCoA aneurysm clipping than after coiling. The causes and characteristics of postoperative cerebral ischemia after PCoA clipping and coiling are different; therefore, treatment should be selected accordingly.

## Background

Posterior communicating artery (PCoA) aneurysms are common and represent approximately 50% of all internal carotid artery aneurysms [1]. Surgical clipping and endovascular coiling are the most common treatments, and both share cerebral ischemia as a potential complication [2–4], which has been associated with poor outcomes [5]. The incidence of postoperative cerebral ischemia is higher with PCoA aneurysms than at other intracranial locations [6, 7]. Reported overall incidence rates of postoperative cerebral ischemia after aneurysm clipping or coiling range from 2.9 to 61% because of interstudy differences in patient, aneurysm, and treatment characteristics and definition of cerebral ischemia [8–10]. Therefore, the studies are difficult to compare, and their findings cannot be generalized.

This study examined cerebral ischemia in patients who underwent clipping or coiling of PCoA aneurysms by the same surgical team. Incidence of ischemia; characteristics of patients, aneurysms, and ischemia; and outcomes were compared between clipping and coiling groups using uniform definitions and evaluation methods. To the best of our knowledge, this is the first study to perform a detailed analysis of cerebral ischemia in PCoA aneurysm patients.

## Methods

### Patients

Consecutive patients with at least one PCoA aneurysm who underwent surgical clipping or endovascular coiling in our institution from January 2017 to December 2019 were retrospectively reviewed. Clinical data were recorded, including age, sex, history of the disease, laboratory, and imaging examination results, surgical details, and outcome. Patients with giant aneurysms (maximum diameter > 25 mm), severe medical comorbidities (e.g., preoperative cerebral ischemia), and incomplete data were excluded.

#### Treatment

Patients were treated with clipping or coiling by a neurosurgeon or interventionalist from the same surgical team based on the patient’s condition and wishes. Precision procedures were performed using intraoperative neurophysiological monitoring to guide intraoperative decision-making. Clipping was performed using the standard pterional approach. Subarachnoid hematoma removal was performed in patients with ruptured aneurysms. Temporary parent artery clipping was used prior to aneurysm clipping as necessary. Complete aneurysm isolation and arterial flow through the parent and penetrating arteries were confirmed after aneurysm clipping using intraoperative indocyanine chloride angiography.

For the coiling, patients with unruptured aneurysms usually require no less than 3 days of dual antiplatelet therapy before endovascular treatment. Furthermore, these patients could be treated endovascularly only if the platelet aggregation rate was below 30% of the initial value. For embolization of ruptured aneurysms, we use the protocol to give tirofiban intravenous pumping after stent placement. A dual antiplatelet loading dose replacement of 300 mg each of clopidogrel and aspirin was given for 24 h postoperatively; followed by a half-dose of tirofiban for 2–4 h of maintenance; followed by a regular dose of dual antiplatelet therapy. Coiling was performed via a transfemoral approach. After femoral artery puncture was performed using the modified Seldinger method, systemic heparinization was initiated. The systemic heparinization protocol for endovascular therapy is: a starting dose of 60–80 IU/kg, followed by an additional half of the initial dose after 1 h and an additional 500–1000 IU every hour afterwards. Appropriate catheters and coils were selected based on aneurysm morphology, size, and aspect ratio (height-to-neck width). Stent- or balloon assistance was used when necessary. After coil occlusion, complete occlusion and arterial flow through the parent and penetrating arteries were demonstrated using intraoperative angiography. All patients were admitted to the intensive care unit after the procedure and were transferred to the general ward the following day, provided that cranial computed tomography (CT) demonstrated no significant abnormalities.

### Evaluation and follow-up

Patients diagnosed with aneurysmal subarachnoid hemorrhage in our institution undergo cranial CT and computed tomography angiography (CTA) in the emergency department. Those with incidentally found or suspected unruptured aneurysms undergo digital subtraction angiography (DSA) for confirmation. Cranial CT is performed at least once within the first seven days after surgery (usually day 1) to screen for intracranial hemorrhagic and ischemic complications. Further brain magnetic resonance imaging is performed in patients with suspected ischemic complications.

Modified Fisher grade before treatment was assessed in patients with aneurysmal subarachnoid hemorrhage. Cerebral ischemia was clinically assessed all before treatment and at discharge using the National Institutes of Health Stroke Scale (NIHSS). Modified Rankin Scale (mRS) score was determined at discharge and six months after discharge. After reviewing the corresponding clinical and imaging data, two independent professionals performed all assessments. Any disagreements were resolved by a third professional who made the final decision.

### Statistical analysis

Statistical analyses were performed on a per-patient or per-aneurysm basis using SPSS software version 26 (IBM Corp., Armonk, NY, USA). Continuous data with a normal distribution are expressed as means with standard deviation. Continuous data with a skewed distribution are expressed as medians with an interquartile range (IQR). Categorical data are expressed as numbers with percentages. Comparisons between the clipping and coiling groups were performed using the chi-square test, Fisher’s exact test, Student’s t-test, or Kruskal–Wallis one-way analysis of variance as appropriate. *P* ≤ 0.05 was considered significant. Graphing was performed using Visio and Excel software (Microsoft, Redmond, WA, USA).

## Results

### Patient and aneurysm characteristics

**Fig. 1:**
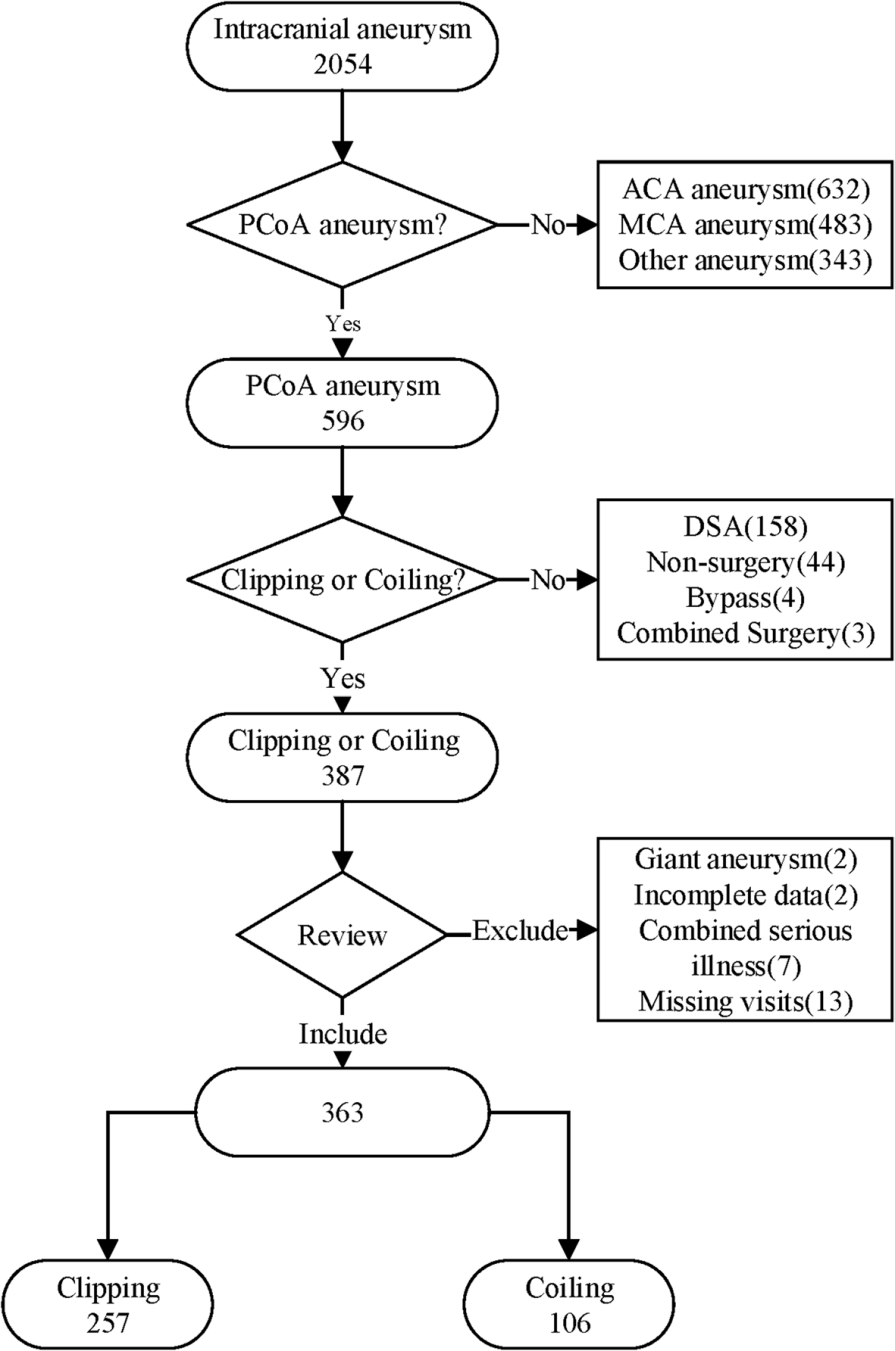
Flow chart of patient collection

**Fig. 2:**
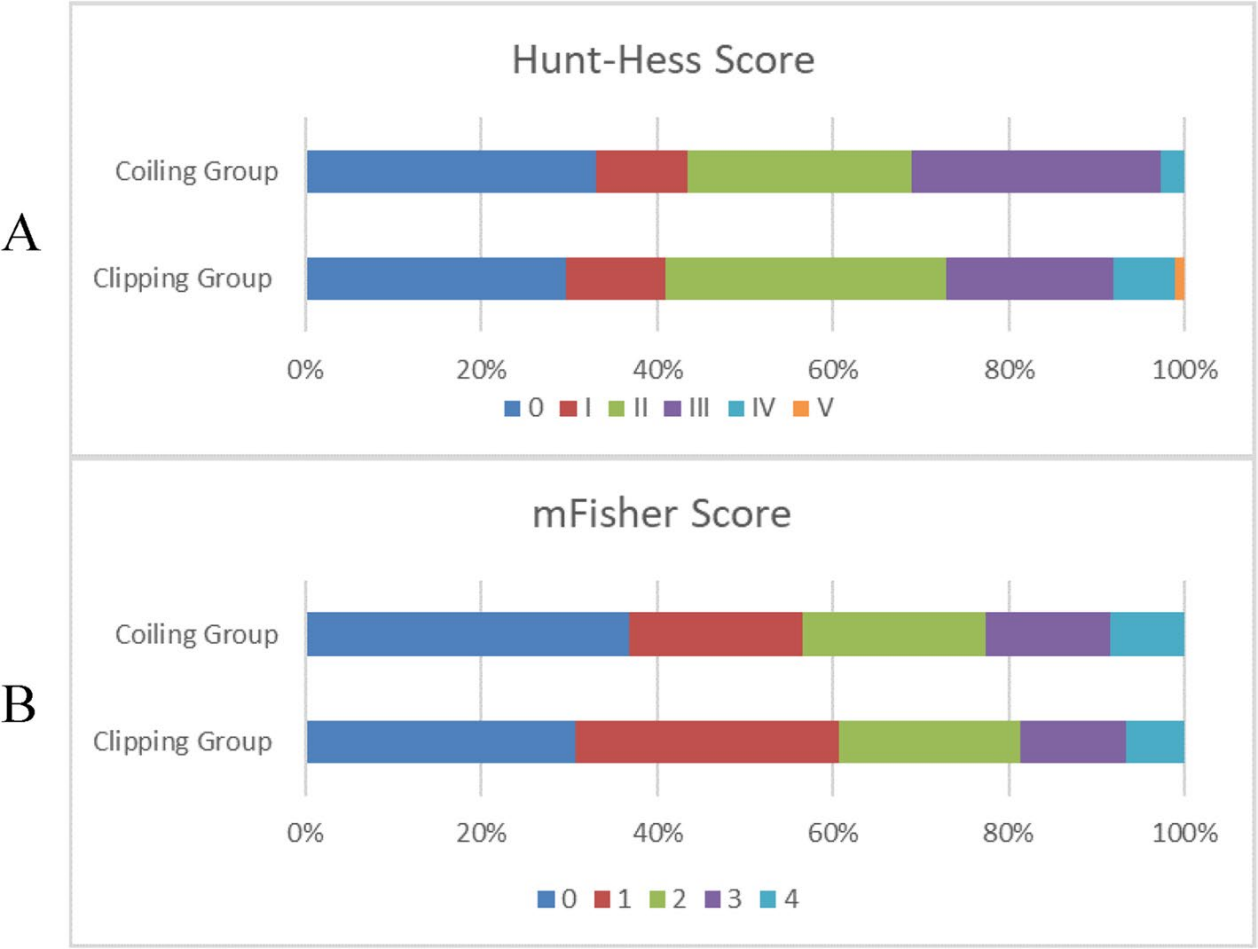
The distribution of Hunt-Hess scores and mFisher scores between the two groups. **A** The distribution of Hunt-Hess scores; **B** The distribution of mFisher scores *Grade 0 is for unruptured aneurysms

Among the 2054 aneurysms treated during the study period, 353 patients with 363 PCoA aneurysms were included for analysis (Fig. 1). Ten patients had bilateral PCoA aneurysms and underwent a second-stage craniotomy, so their two procedures were recorded separately. Clipping and coiling were performed on 257 and 106 PCoA aneurysms, respectively. Patient and aneurysm characteristics and clinical and imaging data are summarized in Table 1. Age, gender, body mass index, and comorbidities (hypertension and diabetes) did not significantly differ between the clipping and coiling groups. The distribution of patients stratified by Hunt–Hess and modified Fisher grades according to the group is shown in Fig. 2 A and B. Although, it appears from the graph that more ruptured and high-grade aneurysms were selected for clip treatment, there was no statistical difference in the Hunt–Hess grade distribution between the two groups. Similarly, there was no statistical difference in the modified Fisher grade distribution between the two groups. There were no significant differences in aneurysm characteristics, including aneurysm size, side, aspect ratio, and whether multiple aneurysms were multiple between the two groups. The overall incidence of ipsilateral oculomotor nerve palsy was 20.39% (74 of 363 aneurysms), even though aneurysms close to or adherent to the nerve were frequently encountered. In the patients who experienced ipsilateral oculomotor nerve palsy, the proportion of patients who underwent clipping was significantly higher than that of those who underwent coiling (85.14% vs. 14.86%; *p* = 0.002).

**Table 1:** Patients’ total characteristics and comparison between the two groups

	Total (*n*=363)	Clipping (*n*=257)	Coiling (*n*=106)	*P*
**Age(y/rs)**	59.04 ± 9.27	58.87 ± 9.12	59.46 ± 9.65	0.254
**Female**	276(78.19%^a^)	198(77.04%)	78(73.58%)	0.483
**Hypertension**	199(56.37%^a^)	148(57.59%)	51(48.11%)	0.099
**Diabetes**	23(6.52%^a^)	14(5.45%)	9(8.49%)	0.279
**Hunt-Hess Score (M, IQR)**	2(3)	2(3)	2(3)	0.755
**mFisher Score (M, IQR)**	1(1)	1(1)	1(1)	0.995
**Pre-NIHSS ** **(M, IQR)**	0(7.25)	0(6.75)	1(7.75)	0.550
**Parent Arteries**				0.715
ICA	73(20.11%)	51(19.84%)	22(20.75%)	
ICA-PCoA	267(73.55%)	188(73.15%)	79(74.53%)	
PCoA	23(6.34%)	18(7.00%)	5(4.72%)	
**Aneurysm Features**				
Unruptured	112(30.85%)	76(29.57%)	35(33.02%)	0.410
Left side	201(55.37%)	142(55.25%)	59(55.66%)	0.943
Neck Diameter	4.82 ± 3.79	4.67 ± 2.36	4.62 ± 1.95	0.481
Top Diameter	6.55 ± 4.16	6.32 ± 3.11	6.42 ± 3.26	0.649
T/N Ratio	1.46 ± 0.66	1.46 ± 0.61	1.48 ± 0.75	0.803
Intraoperative rupture	9(2.48%)	9(3.50%)	0(0%)	0.064
**Fetal PCoA**	22(6.06%)	19(7.39%)	3(2.83%)	0.098
**Paralysis of III nerve**	74(20.39%)	63(24.51%)	11(10.37%)	0.002^a^
**Complications**				
Cerebral Infarction	72(19.83%)	60(23.35%)	12(11.32%)	0.029^**^
Hydrocephalus	24(6.61%)	19(7.39%)	5(4.72%)	0.342
Hemorrhage	9(2.48%)	7(2.72%)	2(1.88%)	1
Intracranial infections	2(0.05%)	2(0.78%)	0	1
**Post-NIHSS ** **(M, IQR)**	0(9.00)	1(12.00)	0(1.75)	0.000^**^
**mRS Score**				
≥2 at Discharge	108(29.75%)	92(35.80%)	16(15.09%)	0.000^**^
≥2 at Follow-up	49(13.50%)	40(15.56%)	9(8.49%)	0.073

** *p* < 0.05, the differences were statistically significant

^a^ The total number of patients was 353 cases

### Postoperative cerebral ischemia

Postoperative cerebral ischemia was the most common postoperative complication (19.83%). Incidence of postoperative cerebral ischemia (23.35% vs. 11.32%, *p* = 0.029) and mean NIHSS score at discharge (1 vs. 0, *p*∴0.001) were higher in the clipping group. The areas of postoperative cerebral ischemia and ischemia manifestations are summarized according to the group in Table 2. The most common area of ischemia in the clipping group was the genu and posterior limb of the internal capsule, which are supplied by the anterior choroidal artery (AChA) and PCoA. In the coiling group, the most common area of ischemia was the centrum semiovale. Half of the patients experienced silent infarction, and the other half presented with various symptoms (transient hemiparesis in 27, consciousness disorder in 10, and aphasia in 6).

**Table 2:** Comparison of postoperative cerebral infarction distribution and symptoms between patients in the clipping and coiling groups

	Infarct location	Clipping	Coiling
**Territory**			
ICA	Cerebral hemispheres	3	
	Hemisphere excluding occipital lobe	2	
AChA	Hind limb of internal capsule	21	2
ACA	Frontal lobe	2	
	Caudate nucleus head	4	
MCA	Temporoparietal lobe	1	
	Globus Pallidus	1	
PCA	occipital lobe	3	1
	thalamus	1	
PCoA	Internal capsule knee	20	2
Unknown origin	Disseminate; Centrum semiovale	8	10
**Clinical presentation**			
Hemiparesis		23	4
Disorders of Consciousness		9	1
Aphasia		6	
Asymptomatic		28	8

*ICA* internal carotid artery, *AChA* anterior choroidal artery, *ACA* anterior cerebral artery, *MCA* middle cerebral artery, *PCA* posterior cerebral artery, *PCoA* posterior communicating artery

Subanalyses were performed in both groups. In the clipping group, the mean age was significantly higher in patients who experienced cerebral ischemia than in those who did not; however, this was not the case in the coiling group (Table 3). Modified Fisher grade and incidence of fetal PCoA were significantly higher in patients who experienced postoperative cerebral ischemia in the coiling group but not the clipping group.

**Table 3:** Comparison of the characteristics of aneurysms and patients with and without ischemia and in the clipping and coiling groups

	Clipping (n = 257)		*P*	Coiling (106)		*P*
	Ischemia (60)	Normal (197)		Ischemia (12)	Normal (94)	
**Age(y/rs)**	60.9 ± 8.21	58.3 ± 9.31	0.048*	61.3 ± 8.33	59.2 ± 9.82	0.479
**Female**	51(85.0%)	147(74.6%)	0.094	7(58.3%)	71(75.5%)	0.203
**Hypertension**	39(65.0%)	109(55.3%)	0.180	7(58.3%)	44(46.8%)	0.452
**Diabetes**	5(8.3%)	9(4.6%)	0.261	2(16.7%)	7(7.4%)	0.281
**Pre-NIHSS (M, IQR)**	2(10.0)	0(4.5)	0.018*	5(12.3)	0(6.5)	0.008*
**Hunt-Hess Score (M, IQR)**	2(2)	2(2)	0.072	2(1)	2(3)	0.080
**mFisher Score (M, IQR)**	1(2)	2(2)	0.072	2(2.5)	1(1)	0.030*
**Parent Arteries**			0.100			0.685
ICA	17(28.3%)	34(17.3%)		3(25.0%)	19(20.2%)	
ICA-PCoA	41(68.3%)	147(74.6%)		9(75.0%)	70(74.5%)	
PcoA	2(3.3%)	16(8.4%)		0(-)	5(5.3%)	
**Aneurysm Features**						
Left side	33(55.0%)	109(55.3%)	0.964	6(50.0%)	53(56.4%)	0.675
Neck Diameter	4.66 ± 2.91	4.68 ± 2.17	0.957	4.55 ± 3.05	5.20 ± 1.77	0.278
Top Diameter	5.62 ± 2.18	6.03 ± 3.17	0.084	6.19 ± 5.30	6.45 ± 2.95	0.798
N/T Ratio	1.36 ± 0.57	1.49 ± 0.62	0.115	1.01 ± 0.50	0.82 ± 0.46	0.181
**Fetal PCoA**	5(8.3%)	14(7.1%)	0.751	2(16.7%)	1(1.1%)	0.002*

*ICA* internal carotid artery, *AChA* anterior choroidal artery, *ACA* anterior cerebral artery, *MCA* middle cerebral artery, *PCA* posterior cerebral artery, *PCoA* posterior communicating artery

* *p* < 0.05, the differences were statistically significant

### Prognosis and follow-up

**Fig. 3:**
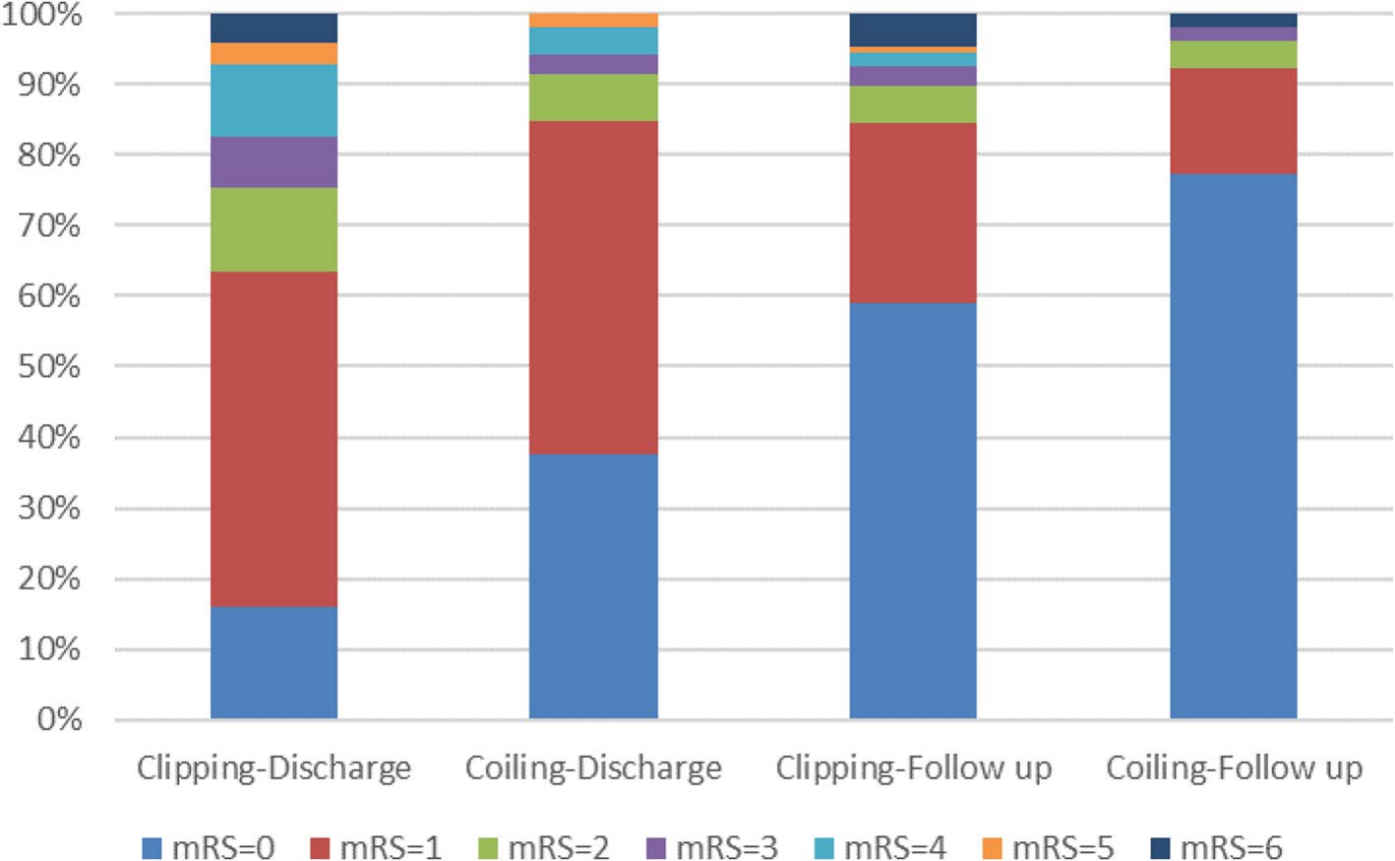
The distribution of mRS between the two groups at discharge and six months of follow-up

We will grade the management according to the NIHSS score for the ischemic event, with enhanced antiplatelet and lipid-regulation treatment for small-vessel lesions and early rehabilitation interventions; large-vessel lesions require urgent surgical management. The vast majority of cerebral ischemia in this study was caused by small vascular lesions, and most recovered well. Overall perioperative mortality was 2.75% (10/363 patients). All deaths occurred in the clipping group. All patients followed up three months after discharge; however, 13 patients (3.46%) were lost at the 6-month follow-up, mainly in the clipping group (*n* = 9). Figure 3 shows the distribution of mRS scores at discharge and six months after discharge according to the group. The proportion of patients with an mRS score ≥ 2 was significantly higher in the clipping group than the coiling group at discharge (35.80% vs. 15.09%; *p* < 0.05) but not six months after discharge (15.56% vs. 8.49%; *p* > 0.05). Four patients died during follow-up, two in each group.

## Discussion

This detailed analysis and comparison of cerebral ischemia after surgical clipping and endovascular coiling of PCoA aneurysms demonstrated that the incidence of postoperative cerebral ischemia was higher in patients who underwent clipping than in those who underwent coiling. Although mRS and NIHSS scores were worse in the clipping group at discharge, the differences were not significant six months later. Subgroup analysis of the clipping group patients showed that the incidence of ischemia was significantly higher in those older and had higher preoperative NIHSS scores. In the coiling group, ischemia incidence was also higher in those with high preoperative NIHSS scores; however, preoperative modified Fisher grade and the presence of a fetal PCoA were associated with ischemia incidence as well. The causes and characteristics of postoperative cerebral ischemia after clipping and coiling appear different. Therefore, treatment should be selected accordingly.

### Incidence of postoperative cerebral ischemia

Our study’s overall incidence of postoperative cerebral ischemia after PCoA aneurysm treatment was 19.8%, which is slightly lower than previously reported rates [8, 9, 11]. One reason may be that we used CT to define cerebral ischemia, which is less sensitive than magnetic resonance imaging. In addition, some previous studies included ischemia that resulted from brain tissue damage caused by surgical access in their definition of postoperative cerebral ischemia; these patients usually did not show any symptoms after the edema resolved [11]. In contrast, we did not. Furthermore, we routinely use intraoperative neurophysiological monitoring when clipping or coiling PCoA aneurysms [12].

Our study found that the incidence of postoperative cerebral ischemia was higher in patients who underwent clipping, which agrees with several previous studies [2, 3, 13]. In a recent study that specifically investigated ruptured PCoA aneurysms, the incidence of postoperative cerebral ischemia was higher in the clipping group than in the endovascular group (24.3% vs. 11.0%) [14]. However, the groups significantly differed in age and gender, which may have influenced the results [15]. Our study groups did not significantly differ. We believe that the difference in ischemia incidence between clipping and coiling is primarily explained by injury or errant clipping of perforating vessels near the aneurysm during surgical clipping [7, 16, 17]. Furthermore, in the clipping group, the incidence of ischemia was higher in older patients, possibly because of worse collateral flow or worse ability to autoregulate flow in older patients.

### Causes of postoperative cerebral ischemia

Cerebral ischemia after intracranial aneurysm treatment may occur because of vascular occlusion owing to various causes or, in patients with aneurysmal subarachnoid hemorrhage, vasospasm caused by blood in the subarachnoid space [18, 19]. First, patients with aneurysms treated by clipping or coiling are at risk for postoperative cerebral ischemia due to vascular occlusion [20]. The AChA and thalamic tubercle artery are penetrating arteries near PCoA aneurysms that arise from the C6 segment of the internal carotid artery and PCoA, respectively [21, 22]. Therefore, the most common site of cerebral ischemia after clipping PCoA aneurysms is the basal ganglia region, supplied by the anterior communication artery (ACoA) and the thalamic tubercle artery [16, 17, 23]. The findings of our study agree: that 80% of postoperative cerebral infarcts occurred in the genu and posterior limb of the internal capsule, which the AChA and thalamic tubercle artery supply. We found that postoperative cerebral ischemia due to injury or errant clipping of perforating arteries was more common than in the coiling group. We believe it may be because clipping is more likely to injure small perforating vessels than coiling, and postoperative antiplatelet therapy, administered to coiling group patients who also underwent stent placement, is an effective prophylaxis against ischemia. However, for PCoA aneurysms in patients with a fetal PCoA, we recommend clipping over coiling.

Blood in the subarachnoid space resulting from aneurysmal rupture causes vasospasm of cerebral arteries that may lead to vascular stenosis or occlusion [8, 15, 24]. Cerebral infarcts caused by vasospasm are usually luminal, disseminated, and most often located in the watershed [8, 18]. In our study, postoperative ischemia occurred in conjunction with treatment in 72 aneurysms (19.83%); among these, infarcts were disseminated and primarily located in the centrum semiovale in 18 (25%). Interestingly, disseminated ischemia was the most common type of cerebral ischemia in the coiling group, and the proportion of patients with disseminated ischemia was significantly higher in the coiling group than in the clipping group (83.33% vs. 13.33%). We believe that subarachnoid hematoma removal performed during clipping procedures may be responsible for this finding [24, 25]. It may also explain why a highly modified Fisher grade was significantly associated with postoperative ischemia in the coiling group but not the clipping group. Alternatively, vasospasm caused by vessel stimulation and induction of microemboli during endovascular coiling may have caused centrum semiovale infarctions in the coiling group [26, 27], as demonstrated in previous studies. Both diagnostic angiography and endovascular treatment of unruptured aneurysms can cause postprocedural lacunar infarction [28, 29].

### Outcome of postoperative cerebral ischemia

Overall, 36 patients (9.92%) exhibited cerebral ischemia symptoms, comparable to previously reported rates [3, 30]. The most common symptoms were transient hemiparesis and impaired consciousness; hemiparesis was permanent in only two patients (1.38%). Most symptoms were transient because they were presumably caused by edema that compressed the internal capsule; such symptoms gradually improve as the edema subsides [31, 32]. Therefore, although the proportion of patients with an mRS score ≥ 2 was significantly higher in the clipping group than in the coiling group at discharge, the difference was not significant six months later. Although we did not identify patients with asymptomatic cerebral ischemia in this study, it is clear that silent ischemic events in the deep white matter are not genuinely silent [27]: in-depth neuropsychological and cognitive testing usually reveals deficits in patients with an otherwise regular neurological examination [33].

### Limitations

This study had several limitations. First, the inherent limitations of retrospective studies are well-known. Second, evaluator bias may have been present in the imaging study assessments, and inter- and intraobserver reliability was not examined. Third, neuropsychological evaluations were not performed; therefore, patients considered asymptomatic may not have been genuinely asymptomatic. These limitations can be overcome in future well-designed prospective studies.

## Conclusion

The incidence of postoperative cerebral ischemia was higher after PCoA aneurysm clipping than after coiling. The most common site of ischemia was the basal ganglia region. Although the proportion of patients with an mRS score ≥ 2 at discharge was significantly higher in the coiling group, the difference was insignificant six months later. The causes and characteristics of postoperative cerebral ischemia after PCoA clipping and coiling are different; therefore, treatment should be selected accordingly. In patients with high modified Fisher grade and those with a fetal PCoA, we recommend clipping over coiling; however, coiling is recommended in older patients.

## Data Availability

The datasets used and/or analysed during the current study available from the corresponding author on reasonable request.

## Abbreviations

ACA: anterior cerebral artery
AChA: anterior choroidal artery
ACoA: anterior communication artery
MCA: middle cerebral artery
CT: computed tomography
CTA: computed tomography angiography
DSA: digital subtraction angiography
ICA: internal carotid artery
IQR: interquartile range
mRS: modified Rankin Scale
NIHSS: National Institutes of Health Stroke Scale
PCA: posterior cerebral artery
PCoA: posterior communicating artery
